# MiR-9 Promotes Apoptosis *Via* Suppressing SMC1A Expression in GBM Cell Lines

**DOI:** 10.2174/2213988501711010031

**Published:** 2017-07-31

**Authors:** Yong Zu, Zhichuan Zhu, Min Lin, Dafeng Xu, Yongjun Liang, Yueqian Wang, Zhengdong Qiao, Ting Cao, Dan Yang, Lili Gao, Pengpeng Jin, Peng Zhang, Jianjun Fu, Jing Zheng

**Affiliations:** 1 Shanghai Key Laboratory of New Drug Design, School of Pharmacy, East China University of Science and Technology, Shanghai 200237, China;; 2 Center for Medical Research and Innovation, Shanghai Pudong Hospital, Fudan University Pudong Medical Center, 2800 Gongwei Road, Pudong, Shanghai 201399, China.

**Keywords:** GBM, miR-9, SMC1A, U87, U251, apoptosis

## Abstract

**Objective:**

Glioblastomas multiforme (GBM) is the most malignant brain cancer, which presented vast genomic variation with complicated pathologic mechanism.

**Method:**

MicroRNA is a delicate post-transcriptional tuner of gene expression in the organisms by targeting and regulating protein coding genes. MiR-9 was reported as a significant biomarker for GBM patient prognosis and a key factor in regulation of GBM cancer stem cells. To explore the effect of miR-9 on GBM cell growth, we over expressed miR-9 in U87 and U251 cells. The cell viability decreased and apoptosis increased after miR-9 overexpression in these cells. To identify the target of miR-9, we scanned miR-9 binding site in the 3’UTRs region of expression SMC1A (structural maintenance of chromosomes 1A) genes and designed a fluorescent reporter assay to measure miR-9 binding to this region. Our results revealed that miR-9 binds to the 3’sUTR region of SMC1A and down-regulated SMC1A expression.

**Result:**

Our results indicated that miR-9 was a potential therapeutic target for GBM through triggering apoptosis of cancer cells.

## 1 INTRODUCTION

Glioblastoma multiforme (GBM) is a grade IV astrocytoma which is the most common and malignant subset of brain tumors [1]. Treatment for glioblastoma patients is mainly surgery in combination with fractionation radiotherapy with concomitant and adjuvant administration of chemotherapy, like temozolomide [2]. GBM patients receive combining treatment of radiotherapy presented a better prognosis than the group receiving radiotherapy only after surgery, and the median survival is 14.6 months [2, 3]. Unfortunately, nearly half of all glioblastoma patients carry an unmethylated MGMT promoter which responded poorly to temozolomide chemotherapy [4]. Thus, revealing the new target in cellular survival and apoptosis resistance will benefit drug development for improving the treatment. Although much is known about the mechanism on GBM survival signaling, there is limited knowledge of apoptotic mechanism of GBM from bench to bedside. Generally, specific signaling pathways cause apoptosis which are often deregulated in cancer. An apoptotic regulation protein BCL-2L12 was found to be overexpressed in nearly all GBMs, and a bunch of X-linked inhibitor of apoptosis (XIAP) are emerging as potential therapeutic targets for GBM [5, 6]. Besides coding genes, non-coding genes as microRNAs are also highly valued target for cancer therapy.

MiRNAs (microRNAs) are small non-coding RNAs (19–25 nucleotides) and negative post-transcriptional regulator of gene expression [7]. Computational research revealed that 149 microRNAs were involved in GBM tumorgenicity via RNA-RNA interaction with expression genes [8]. Further study proved that miR-21 was a vital negative apoptotic factor in GBM by suppressing caspase pathway, which made it a plausible therapeutic target [9]. High throughput gene expression screening of clinical samples demonstrated that miR-9 is an important marker in the prognosis of GBM which presents highly expressed in advanced WHO grades in glioma patients group than in poor prognosis group [10]. Accumulating research in cancer cells demonstrated that miR-9 was a tumor suppressor in gastric, ovarian and breast cancer while targeting various mRNA [11, 12]. In GBM cells with EGFR mutation, which is occurring in large proportion of patients, miR-9 is identified as a tumor suppressor which is regulated by FOXP1 [13]. Recently, Efroni *et al.* reported that the hsa-miR-9 induced decrease in migration and invasion of GBM cells, is directly mediated through MAPKAP signaling [14]. These findings strongly indicated that miR-9 is an interesting therapeutic target for GBM and it suppressed cancer cell growth through multiple pathways.

In previous study, we demonstrated that down-regulation of SMC1A inhibited GBM cell growth by G2/M arresting [15]. Accumulating evidences proved that SMC1A was also involved in various cellular functions including growth, migration and apoptosis. Sun *et al.* found down-regulation of SMC1A inhibited lung adenocarcinoma cells A549 and H1299 cells by G0/G1 arrest, meanwhile, the apoptotic pathway was also activated [16]. Moreover, SMC1A was proved to play an important role in colorectal cancer metastasis by stimulating inflammatory mediators [17]. Integrated computational analysis suggested that SMC1A was a potential target of miR-9 [18-20]. Here we reported that miR-9 overexpression triggered the apoptosis of GBM cell lines and SMC1A was a direct target of miR-9.

## 2 MATERIALS AND METHODS

### 2.1 Cell Culture

The human glioblastoma cell lines U87 and U251, were purchased from the American Type Culture Collection (ATCC), and cultured in α-MEM media (containing L-glutamine), supplemented with 10% fetal bovine serum (FBS) and 1% Antibiotic-Antimycotic (AA). All cultured cells were maintained at 37^o^C in a humidified atmosphere containing 5% CO_2_.

### 2.2 Construction of miR-9 Expression Lentivirus Vectors

To generate lentivirus expressing mature miRNA of miR-9 the pre-miRNA sequence (AGT ATG TCG ATC TAT TGG TTT CT) and universal control (a sequence which does not match any human genes) were synthesized and linked into a vector. The sequences were cloned into the HpaI and XhoI sites of the pLKD-GFP (Neuronbiotech, Shanghai, China) to generate pLKD- GFP-miR-9 or pLKD-GFP-Ctr, respectively. Viral shRNA of SMC1A was obtained as previously described [15].

### 2.3 Lentivirus Production

To produce lentiviral vector, the plasmids encoding miR-9, SMC1A shRNA or control were individually mixed with plasmids, pHelper1.0 and pHelper 2.0 (virus packaging helper plasmid) and co-transfected into HEK293T cells with lipofectamine 3000, according to the manufacture’s instruction (Invitrogen). After 48h incubation, the culture supernatants containing virus were harvested and ultra-centrifuged. The virus titers of each preparation were determined. To perform lentiviral infections, the U87 and U251 cells were plated at 40% - 50% confluence and incubated overnight (16 h). On the day of infections, the culture medium was replaced by the appropriate addition of virus and incubated at 37^o^C for 12~16h, followed by a medium replacement. 24~36 hours later, infected cell populations were observed under fluorescent microscopy. After 5 days of selection, shRNA knockdown efficiency was determined by quantitative real-time RT-PCR and western blot analysis.

### 2.4 RNA Extraction and Quantitative Real-Time PCR

To analyze miR-9 and SMC1A expression level, approximately 1.0×10^6^ U87 or U251 cells (uninfected or infected cells) were seeded into 6-well culture plates, respectively. Cells of each group were harvested after cultured for 72 h. Small RNAs (~200 nt) were isolated with mirVanaTM PARIS TM Kit (Ambion) according to the manufacturer’s instructions. For RT reactions, 1 µg of small RNA was used for reverse transcription with RT primers at 37 ^o^C for 60 min and a final incubation at 95^o^C for 5 min. MicroRNA qRT-PCR was carried out by using the miScript SYBR Green PCR kit (Qiagen) on an Applied Biosystems 7000 real-time PCR machine (ABI). The PCR reaction was conducted at 95^o^C for 15 min, followed by 40 cycles of incubation at 94^o^C for 15s, 55^o^C for 30s, and 70^o^C for 30s. Expression level of each miRNA was normalized by against the U6 snRNA levels. Total RNA was extracted from cells with TRIzol reagent (Invitrogen) according to the manufacturer’s instructions. Expression of SMC1A mRNA was detected by qRT-PCR using the standard SYBR Green RT-PCR Kit (Takara) according to the manufacturer’s instruction. Briefly, the cDNA was synthesized using the RevertAid First-Strand cDNA Synthesis Kit (Fermentas, Lithuania), according to the manufacturer’s protocol. The cDNA was used as the template in an iQTM SYBR Green Supermix (Bio-Rad, Her-cules, CA) and in triplicate subjected to denaturation at 94^o^C for 1 min and 30 cycles of 94^o^C for 40 sand 60^o^C for 40s, followed by extension at 72^o^C for 6 min. The relative levels of SMC1A mRNA transcripts were normalized to the control GAPDH. Relative gene expression was quantified using the GraphPad Prism 5 software (GraphPad Software, San Diego, CA) and expressed as % of the control. Primers used in microRNA reverse transcription and specific amplification are listed in (Table **1**).

### 2.5 Western Blotting

Cells cultured in 35 mm dishes were lysed in 0.2 ml lysis buffer (0.1% SDS, 1% NP-40, 50 mM HEPES, pH 7.4, 2 mM EDTA, 100 mM NaCl, 5 mM sodium orthovanadate, 40 μM p-nitrophenyl phosphate, and 1% protease inhibitor mixture set I; Calbiochem). Cell lysates were centrifuged at 12,000 rpm for 15 min. The supernatant was collected and denatured. Proteins were separated in 10% SDS-PAGE gel and blotted onto polypropylene difluoride membrane. The blot was blocked for 1.5 h at room temperature in 5% BSA, followed by overnight incubation at 4°C with indicated antibodies. Membranes were rinsed and incubated for 1 h with the correspondent peroxidase-conjugated secondary antibodies. Chemiluminescent detection was performed with the ECL kit (Pierce).

### 2.6 MTT Assay

Cell viability and proliferation were evaluated by the MTT method. Cell viability of U251 with miR-9, SMC1A shRNA or control was assessed by the MTT assay performing at four time points (on day 1, 2, 3 and 4) after successful infection. Briefly, quantification of mitochondrial dehydrogenase activity was achieved via the enzymatic conversion of MTT [3-(4,5-dimethyldiazol-2-yl)-2,5- diphenyltetrazolium bromide] (Sigma-Aldrich) to a colored formazan product. The test cells in exponential growth were plated at a final concentration of 2 × 10^3^ cells/ well in 96-well culture plates for different culture time (1 day, 2 day, 3 day, and 4 day, respectively). MTT (10 μl, 10 mg/ml) was then added. After an additional 4 h of incubation, the reaction was terminated by removal of the supernatant and addition of 100 μl DMSO to dissolve the formazan product. After 0.5 h, the optical density (OD) of each well was measured at 570nm using an ELISA reader (ELx808 Bio-Tek Instruments, USA).

### 2.7 Wound Healing Assay

Lentivirus infected U251 cells were seeded in 12-well plates until they reached 60–70% confluence. After 12 hours, cell monolayer was scraped in a straight line to create a “scratch” with a pipet tip. The cells were washed with growth medium to remove debris and to smooth the edge of the scratch followed by a medium replacement with 1 mL growth medium and incubation for 24 h at 37°C in 5% CO_2_. Images were taken at 0 and 24 hours by placing the 12-well plate under the microscope (Nikon Ti-S automated Inverted) using an automated program to ensure the same area is aligned and photographed.

### Flow Cytometry Test

For flow cytometry determination, U251 cells were harvested with trypsin and fixed with 30min 70% ethanol. Afterwards, cells were treated with RNase for 30min, then incubated with Annexin V-PE in darkness for 30min. Flow cytometry was performed by BD FACS Calibur and data was recorded by FlowJo 7.6.1 and analyzed by GraphPad Prism 5 software.

### 3'UTR Reporter Assay

UTR binding reporter assays were performed in HEK293T cells. pMIR- REPORT vectors harboring 1200bp fragment of SMC1A-3'UTR behind initiated from stop codon with wild type (WT) miR-9 binding sites (2146-2153) or mutated (MUT) miR-9 binding sites were produced by cloning the synthesized fragments into the HindIII and SpeI restriction sites of pMIRREPORT: Cells were infected with miR-9 or negative-control lentiviral vectors under appropriate MOI condition, with pMIRREPORT vectors containing WT or MUT miR-9 binding sites (400 ng) and pRL-SV40 (Promega) expressing Renilla luciferase (400 ng) for normalization. Cells were grown in high-glucose DMEM supplemented with 10% fetal bovine serum, and luciferase activity measurements were performed 48 hours post-transfection using the Dual-Luciferase Reporter Assay System (Promega).

### Statistical Analysis

Data were expressed as mean ± SD. Statistical analysis was performed using SPSS software (Release 11.0, SPSS Inc.). The difference between two groups was analyzed by the Student’s t-test. A value of p < 0.05 was considered as statistical significance.

## RESULTS

### MiR-9 Overexpression Reduced SMC1A Expression in U87 and U251 Cells

**Fig. (1):**
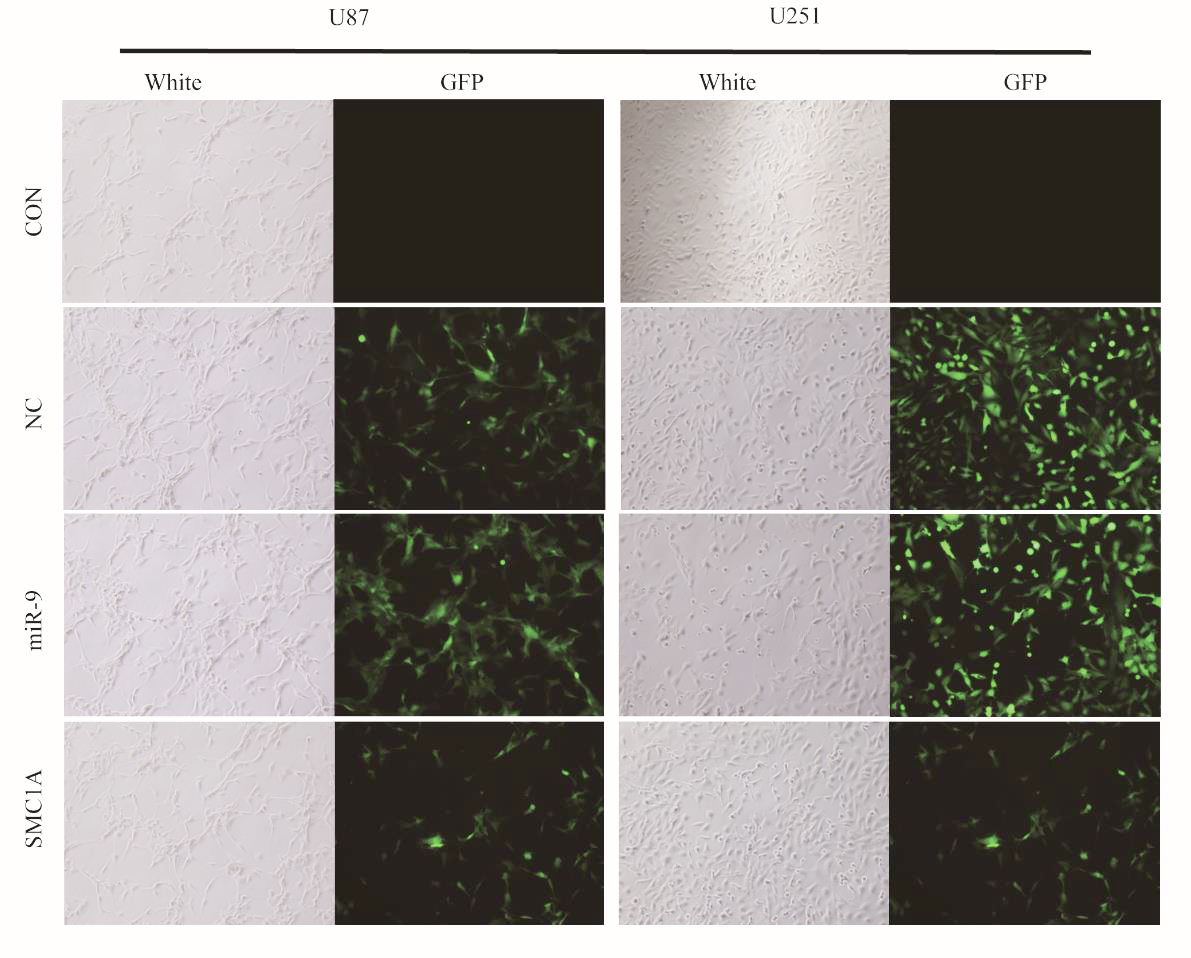
Lentivirus infection of U87 and U251 cells. U87 and U251 cells is infected with Negative Control (NC), miR-9 expression and SMC1A shRNA lentivirus at MOI=10. After 48~72h above 80% of infected cells presented GFP expression. Scale bar represents 400µm.

**Fig. (2):**
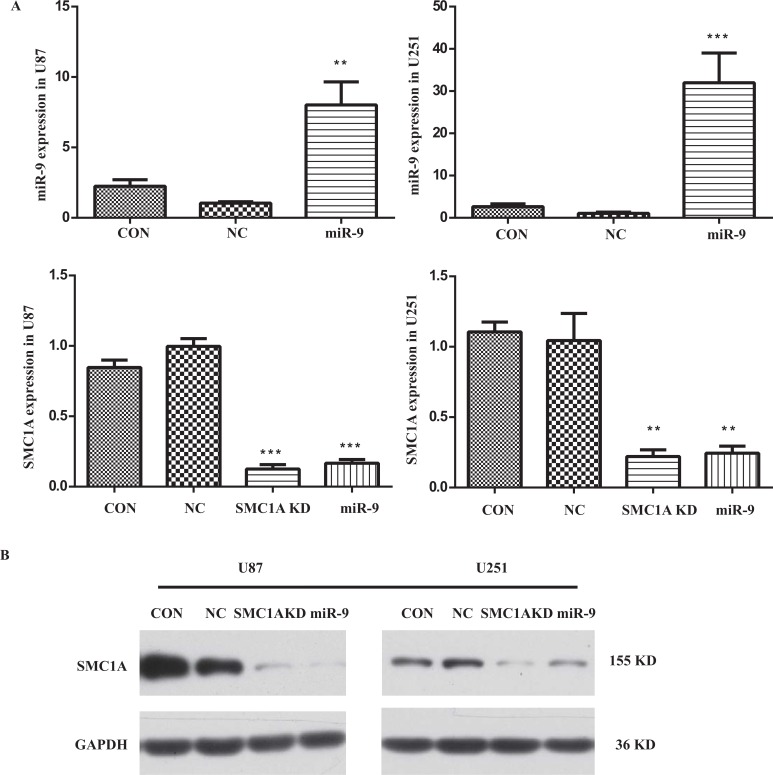
miR-9 and SMC1A expression levels in lentivirus infected U87 and U251 cells.(**A**) miR-9 level was significantly increased after the lentivirus vector infection in both cell lines. SMC1A level was significantly decreased by either miR-9 overexpression or the gene knockdown with RNAi of SMC1A (SMC1A KD, KD for knock down). The result is average of three independent replicates. **indicates significance at p<0.01. ***indicates p<0.005; (**B**) Western blot analysis of SMC1A protein levels either in miR-9 overexpression or in the gene knockdown with RNAi of SMC1A.

To determine the impact of miR-9 on SMC1A expression level, we firstly constructed lentivirus harbored mature miR-9 sequence. We infected U87 and U251 cells with this lentivirus vector at MOI of 10 and performed qPCR and western blotting analysis in the infected U87 and U251 cells (Fig. **1**). The relative miR-9 expression level was normalized to a housekeeping gene U6 in the qPCR test. Results showed that it increased 7 folds in U87 cells and 30 folds in U251 cells after the lentivirus infection. With these high overexpression levels of miR-9, the mRNA level of SMC1A in GBM cells was significantly decreased to 15% which is similar to cells infected by lentiviral SMC1A shRNA. To confirm the down-regulation of SMC1A by miR-9 overexpression, we further performed western blotting analysis. The results showed that SMC1A protein expression decreased dramatically by either miR-9 overexpression or gene knockdown with the SMC1A shRNA in GBM cells (Fig. **2**).

### Inhibition of Cell Growth and Increase in Apoptosis by Overexpression of miR-9

**Fig. (3):**
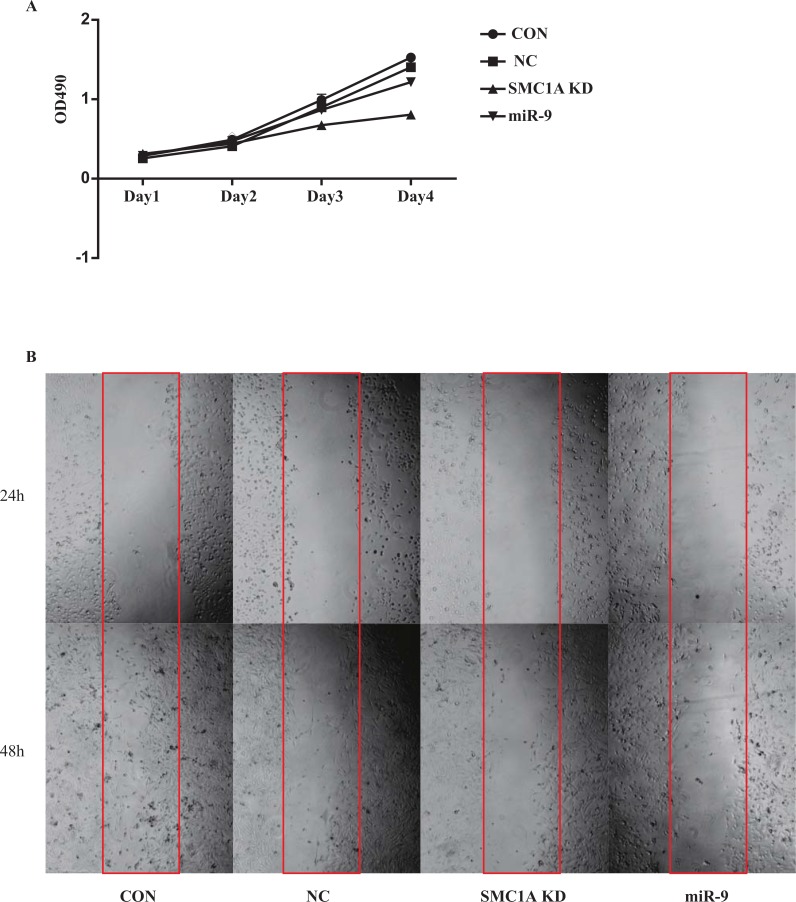
Cell viability of U251 cells. (**A**) MTT test of cells illustrated growth of GBM cells inhibited by SMC1A RNAi and miR-9 overexpression; (**B**) miR-9 overexpression and SMC1A RNAi decreased the wound healing ability of U251 cells.

**Fig. (4):**
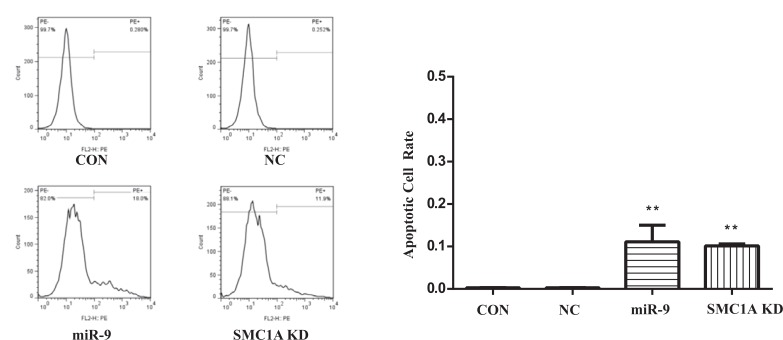
Apoptosis of U251 cells. miR-9 and SMC1AshRNA significantly promoted apoptosis of U251 cells. ** indicate significant variation among miR-9, SMC1A and NC group with p<0.01.

To investigate the effect of miR-9 in GBM cells, U251 cells or U87 cells were infected by negative control, miR-9 and SMC1A shRNA lentivirus vectors respectively. MTT and wound healing assays were used to determine the proliferation and migration of the cells. Results showed that both miR-9 overexpression and SMC1A knockdown significantly decreased the cell growth rate and migration of U251 cells (Fig. **3**). Furthermore, we applied the flow cytometry method to determine the apoptosis rate of both U87 and U251 cells. Results showed that miR-9 overexpression promotes the apoptosis of GBM cells, which is similar to that in the SMC1A knockdown group (Fig. **4**).

### SMC1A Expression was Reduced by Binding of miR-9 to SMC1A 3'UTR

**Fig. (5):**
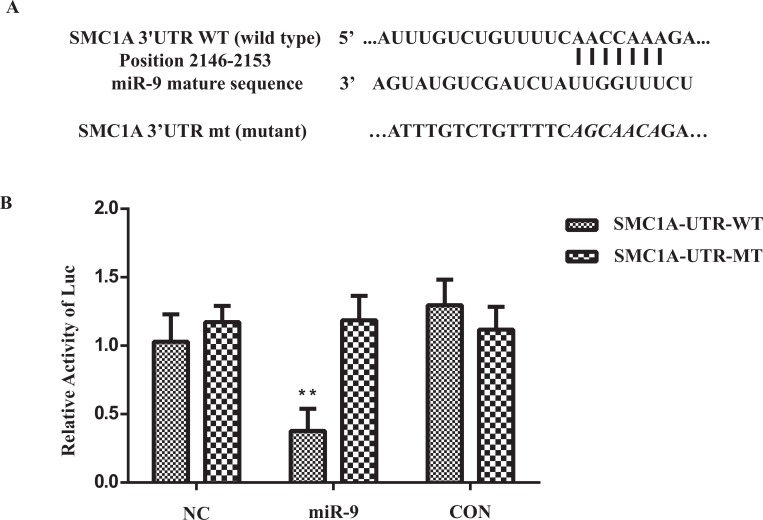
Validation of SMC1A 3’UTR and miR-9 combination. (**A**) The seed sequence for combination of miR-9 and SMC1A 3’UTR. (**B**) miR-9 overexpression showed significant suppression of Luciferase expression which linked to wildtype 3’UTR of SMC1A. **indicates significant variation between miR-9 group versus NC group, p<0.05.

To validate the regulation mechanism of miR-9 on SMC1A expression, we employed a luciferase reporter assay that linked the 3’UTR region of SMC1A to the luciferase gene. The combining sites of SMC1A 3’UTR with miR-9 were predicted by TargetScan [21], PicTar [22], and TargetRank [23]. A sequential modification of 8 nucleotides ‘seed’ region in the 3’UTR of SMC1A was used for mutant vector as a negative control. The HEK293T cells were infected by miR-9 or negative control lentivirus vector at MOI of 1 prior to the luciferase reporter vector transfection. The luciferase intensity decreased significantly miR-9 overexpression, while the mutant group showed no obvious change, indicating that the binding of miR-9 to the wild type SMC1A 3’UTR group is required for the SMC1A transcription and expression. This result also validated the predicted binding site of miR-9 in the 3’UTR region of SMC1A (Fig. **5**).

## DISCUSSION

MiR-9 is an important anti-oncogene in GBM and other cancers such as leukemia, breast cancer, nasopharyngeal carcinoma and hepatocellular carcinoma (HCC). It inhibits various processes as proliferation, metastasis and vascular formation [24-29]. Increase in miR-9 expression in EGFR mutation bearing cell line leads to tumor suppression via direct regulation of FOXP1 [13]. Moreover, miR-9 overexpression also suppressed migration and invasion of GBM cells by inhibiting MAPK signaling activity [14]. In breast cancer cells, MiR-9 targeted ZEB1 gene, inhibited endothelial differentiation pathway and HCC proliferation by suppressing TAZ gene expression. However, our knowledge of effect of miR-9 on cancer apoptosis is limited.

As we previously indicated, when knocking down SMC1A expression in U87 and U251, cell proliferation and clone formation ability were dramatically declined [15]. In addition, in vivo experiment indicated that block of SMC1A impaired tumorgenesis of prostate cancer cells. SMC1A was originally identified as an essential factor for chromosome segregation and DNA repair. Recently, accumulating evidence indicated that cohesin participated in other processes that involved DNA looping, especially, transcriptional regulation [16, 30-33]. Moreover, down regulation of SMC1A in lung adenocarcinoma cells and colorectal cancer cells induced apoptosis [16, 34]. It implied that SMC1A may play a role in apoptosis of GBM cell lines.

In this study, we indicated that miR-9 overexpression induced apoptosis of GBM cells. As a computationally predicted target of miR-9, SMC1A was also verified as an apoptotic inhibitor to GBM cell lines. We then performed luciferase reporter assay to examine the directly binding of miR-9 to the 3”UTR region and its effects upon SMC1A expression in HEK293T cells. We found that both shRNA and miR-9 down regulated SMC1A expression through binding to the 3’UTR region of SMC1A gene, which further blocked cell growth and triggered the apoptosis of the GBM cell lines. Therefore, miR-9 is a potential therapeutic target for development of a new GBM therapy via promoting cancer cell apoptosis. Both artificial oligonucleotides and viral gene therapy that increase miR-9 level in GBM cells could be considered as useful therapeutic approaches.

## CONCLUSION

Taken together, the results obtained in the present study demonstrate that the microRNA miR-9 negatively regulates SMC1A expression in GBM cells which reduces cancer cell growth and increases apoptosis. The mechanism of action is that miR-9 binds to the 3’UTR region and inhibits the transcription and expression of SMC1A. The results indicate that miR-9 is a potential therapeutic target for development of new therapeutics to treat GBM.

## Figures and Tables

**Table 1:** Primers used in miR-9 and SMC1A RT-PCR test.

Gene	Primer	Sequence
GAPDH	GAPDHF	AAGGTCGGAGTCAACGGATT
	GAPDHR	CTCCTGGAAGATGGTGATGG
SMC1A	SMC1A-QF	TCGGA CCATT TCAGA GGTTCA
	SMC1A-QR	TCCTC AGAGT AGACC ATGCT G
miR-9	Mi9RT (for reverse transcription)	CTCAACTGGTGTCGTGGAGTCGG CAATTCAGTTGAG tca tacag
	mi9F	ACACTCCAGCTGGG tcttt ggtta tctag
	mi9R	TGGTGTCGTGGAGTCG
U6	U6F	CTCGCTTCGGCAGCACA
	U6R	AACGCTTCACGAATTTGCGT
